# Age-associated epigenomic heterogeneity in papillary tumors of the pineal region: a multicenter YoungNOA investigation

**DOI:** 10.1186/s40478-026-02411-x

**Published:** 2026-08-28

**Authors:** Lazaros Lazaridis, Xiaomei Zhou, Raphael Bodensohn, Malte Mohme, Julia Onken, Regina von Manitius, Naomi Houedjissin, Tobias Blau, Ilinca Popp, Sarina Agkatsev, Sabine Seidel, Christina Schaub, Maximilian Scheer, Andre Sagerer, Volker Neuschmelting, Davide Tosin, Viktoria Ruf, Michael C. Burger, Christoph Oster, Teresa Schmidt, Benjamin Thiele, Jürgen Hench, Stephan Frank, Martin Stuschke, Laurèl Rauschenbach, Stephan Tippelt, Beate Timmermann, Kathy Keyvani, Clemens Seidel, Andrea Tannapfel, Dorothea Miller, Niklas Thon, Joachim P. Steinbach, Elke Pfaff, Georg Karpel-Massler, Jörg Felsberg, Guido Reifenberger, Marcos Tatagiba, Ghazaleh Tabatabai, Jens Schittenhelm, Christian Thomas, Martin Hasselblatt, Corinna Seliger, Christoph Kleinschnitz, Ulrich Sure, David Capper, Michael Platten, Felix Behling, Michael Müther, Sied Kebir

**Affiliations:** 1 Department of Neurology, University Hospital Knappschaftskrankenhaus Bochum, Ruhr University Bochum, Bochum, Germany; 2 Division of Clinical Neuro-Oncology, Department of Neurology and Center for Translational Neuro- and Behavioral Sciences (C-TNBS), University Medicine Essen, University Duisburg-Essen, Hufelandstr. 55, 45147 Essen, Germany; 3 Department of Radiation Oncology, University Hospital Tübingen, Tübingen, Germany; 4 Department of Neurosurgery, University Medical Center Hamburg-Eppendorf, Hamburg, Germany; 5 Department of Neurosurgery, Charité - Universitätsmedizin Berlin, Berlin, Germany; 6 Department of Neuropathology, Charité - Universitätsmedizin Berlin, Berlin, Germany; 7 Department of Neurosurgery, Medical Faculty, Center of Neuro-Oncology, Heinrich-Heine-University Düsseldorf, Düsseldorf, Germany; 8 Institute of Neuropathology, University Medicine Essen, Essen, Germany; 9 Department of Radiation Oncology, Medical Center-University of Freiburg, Faculty of Medicine, University of Freiburg, Freiburg, Germany; 10 Department of Neurosurgery, University Hospital Heidelberg, Heidelberg, Germany; 11 Department of Neurosurgery, Universität Carl Gustav Carus, Technische Universität Dresden, Dresden, Germany; 12 Department of General Neurosurgery, Center for Neurosurgery, Faculty of Medicine and University Hospital Cologne, University of Cologne, Cologne, Germany; 13 Department of Neurological Surgery, Ulm University Medical Center, Ulm, Germany; 14 Faculty of Medicine, Center for Neuropathology and Prion Research, LMU Munich, Munich, Germany; 15 Goethe University, Dr. Senckenberg Institute of Neurooncology, Goethe University Hospital, Frankfurt Am Main, Germany; 16 German Cancer Consortium (DKTK), Partner Site Essen/Düsseldorf, a Partnership Between DKFZ and University Hospital Essen, Germany, DKFZ-Division Translational Neuro-Oncology at the West German Cancer Center (WTZ), University Medicine Essen, University Duisburg-Essen, Essen, Germany; 17 Medical Oncology, University Hospital Basel, Basel, Switzerland; 18 Laboratory of Translational Immuno-Oncology, Department of Biomedicine, University and University Hospital Basel, Basel, Switzerland; 19 Institute for Pathology and Medical Genetics, University Hospital Basel, Basel, Switzerland; 20 Department of Radiotherapy, University Medicine Essen, University Duisburg-Essen, Essen, Germany; 21 Department of Neurosurgery and Spine Surgery, University Medicine Essen, University Duisburg-Essen, Essen, Germany; 22 Department of Pediatrics III, Center for Translational Neuro- and Behavioral Sciences (C-TNBS), University Hospital of Essen, 45122 Essen, Germany; 23 Department of Particle Therapy, University Hospital Essen, West German Proton Therapy Centre Essen (WPE), West German Cancer Centre (WTZ), 45147 Essen, Germany; 24 German Cancer Consortium (DKTK), 45147 Essen, Germany; 25 Department of Radiotherapy and Radiation Oncology, University Hospital Leipzig, Leipzig, Germany; 26 Institute of Pathology, Ruhr University Bochum, Bochum, Germany; 27 Department of Neurosurgery, University Hospital Knappschaftskrankenhaus Bochum, Ruhr University Bochum, Bochum, Germany; 28 Department of Pediatric Oncology, Hematology and Immunology, Heidelberg University Hospital, Heidelberg, Germany; 29 Institute of Neuropathology, Medical Faculty, Heinrich-Heine-University Düsseldorf, and University Hospital Düsseldorf, Düsseldorf, Germany; 30 Department of Neurosurgery, University Hospital Tübingen, Tübingen, Germany; 31 Department of Neurology and Interdisciplinary Neuro-Oncology, University Hospital Tübingen, Hertie Institute for Clinical Brain Research, Center for Neuro-Oncology, Comprehensive Cancer Center, Eberhard-Karls-University Tübingen, Tübingen, Germany; 32 Department of Neuropathology, Institute of Pathology and Neuropathology, University Hospital Tübingen, Eberhard-Karls-University Tübingen, Tübingen, Germany; 33 Institute of Neuropathology, University Hospital Münster, Münster, Germany; 34 German Cancer Consortium (DKTK), Partner Site Berlin, and German Cancer Research Center (DKFZ), Heidelberg, Germany; 35 Department of Neurology, Medical Faculty Mannheim, Mannheim Center for Translational Neurosciences (MCTN), University of Heidelberg, Mannheim, Germany; 36 Department of Neurosurgery, University Hospital Münster, Münster, Germany; 37 Department of Neurosurgery, Faculty of Medicine, University of Augsburg, Augsburg, Germany; 38 Department of Neurooncology, Centers for Neurology and Integrated Oncology (CIO), University Hospital Bonn, Bonn, Germany

**Keywords:** Papillary tumors of the pineal region, Epigenomic, DNA methylation

## Abstract

**Background:**

Papillary tumors of the pineal region (PTPR) are rare CNS neoplasms with adult and pediatric presentations, but whether age defines distinct molecular biology is unclear.

**Methods:**

We assembled a multicenter retrospective cohort of 86 histologically confirmed PTPR with genome-wide DNA methylation data, comprising 62 adult and 24 pediatric tumors. Molecular subgroup, array platform, sex, and tumor purity were incorporated into multivariable models. Analyses included DNA methylation class assignment, differential methylation, copy-number variation (CNV), epigenetic mitotic-clock scores, methylation-based tumor microenvironment deconvolution, and descriptive survival evaluation.

**Results:**

Adult and pediatric tumors mapped within the established PTPR-A and PTPR-B methylation framework rather than forming age-defined methylation classes. Pediatric tumors were enriched for PTPR-B (22 of 24 tumors [91.7%]) compared with adult tumors (39 of 62 [62.9%]). After adjustment for methylation-based subgroup as well as technical and biological covariates, 2,923 CpG probes were associated with age at a false discovery rate (FDR) threshold below 0.05, and 530 also met the prespecified effect-size threshold. Global methylation summaries were similar between age groups. CNV patterns were dominated by molecular subgroup; adjusted genomic CNV load was not independently associated with pediatric age. In contrast, epiTOC2 intrinsic rate score and the methylation signature represented by the first principal component (PC1) showed age-associated effects independent of molecular subgroup. Methylation-based deconvolution suggested a limited microenvironmental signal, with neutrophil fraction showing the most consistent adjusted association.

**Conclusions:**

Adult and pediatric PTPR share the established PTPR-A/PTPR-B framework. Pediatric tumors, particularly within PTPR-B, showed age-associated DNA methylation differences and higher epigenetic mitotic-clock (epiTOC2) scores in this retrospective cohort. These tissue-level associations do not establish clinical risk or treatment implications and require prospective clinical annotation and orthogonal validation.

**Supplementary Information:**

The online version contains supplementary material available at https://doi.org/10.1186/s40478-026-02411-x.

## Introduction

Papillary tumors of the pineal region (PTPR) were first recognized as a distinct pineal region tumor in 2003 [1] and entered the World Health Organization (WHO) classification of central nervous system (CNS) tumors in 2007 [2]. In the 2021 WHO classification, PTPR remain a circumscribed pineal region tumor assigned CNS WHO grade 2 or 3 depending on histological and immunohistochemical criteria [3]. Its papillary and solid architecture with epithelial-like cytology are described in histopathologic series [4, 5]. Symptoms of increased intracranial pressure are a common clinical presentation in PTPR series [6, 7], whereas local recurrence, cerebrospinal-fluid dissemination, and heterogeneous outcomes have been reported in multicenter and systematic clinical analyses [6–8]. A recent case-series literature review further underscores that treatment consensus remains limited [9]. This clinical heterogeneity has made a more precise understanding of PTPR biology clinically relevant.

Early molecular analyses supported PTPR as a distinct molecular entity [10], and DNA methylation-based classification subsequently established PTPR as a distinct CNS methylation class [11]. Molecular subgrouping studies of pineal tumors and PTPR identified the major PTPR-A and PTPR-B epigenetic subgroups [7, 12]. Copy-number differences between these subgroups support biologic heterogeneity within a tumor type that has often been approached as a single diagnostic entity [7, 10, 12]. Most previous molecular studies have analyzed mixed adult and pediatric cohorts and have primarily focused on defining PTPR as a single molecular entity [7, 12]. Whether adult and pediatric PTPR share the same molecular framework or show age-associated differences in their epigenetic profiles, genomic instability, and tumor microenvironment remains uncertain.

Age is an important determinant of the biology of several CNS tumors. In glioma, age is associated with epigenetic subtype and epigenetic-age patterns [13, 14], whereas medulloblastoma shows age-linked molecular subgroup and structural-variation patterns [15]. Recent pediatric pineal-tumor literature also emphasizes molecular heterogeneity in this anatomic compartment [16]. By contrast, it is not known whether pediatric and adult PTPR represent age-modified presentations of the same molecular disease or whether age is associated with biologically meaningful molecular features.

In this study, we assembled a retrospective multicenter cohort of adult and pediatric PTPR with genome-wide DNA methylation data and clinical annotation. We first asked whether adult and pediatric tumors formed distinct epigenetic entities or instead shared the established PTPR-A and PTPR-B methylation subgroup framework. We then used multivariable models to assess age-associated molecular differences after accounting for subgroup-related confounding, with analyses focused on differentially methylated CpG sites, genomic copy-number variation burden, epigenetic mitotic-clock signatures, and methylation-based cell type deconvolution. Our aim was to define age-associated molecular features of potential biological and clinical relevance in PTPR.

## Methods

### Study design and data sources

We assembled a retrospective cohort of PTPR cases from multiple centers with clinical annotation and genome-wide DNA methylation data. The adult cohort (n = 62) combined internally collected cases with previously published cases from Wu et al. [7] and Capper et al. [11]. For the external adult cases, clinical data and Illumina 450 K or EPIC methylation IDAT files were downloaded from the Gene Expression Omnibus (GEO; accession numbers GSE254031 and GSE90496). The pediatric cohort (n = 24) was not internally accrued; all pediatric cases were obtained from the published Wu et al. PTPR series, with matched clinical annotation and Illumina 450 K or EPIC IDAT files from GEO accession GSE254031.

The internal adult series initially included 25 patients with histopathologically confirmed PTPR diagnosed between 2007 and 2022. These patients were recruited at ten German neuro-oncology centers affiliated with the Young Neuro-Oncological Working Group (YoungNOA) of the German Cancer Society (DKG). The protocol was approved by the lead Ethics Committee of the University Hospital Essen (approval number 20-9431-BO) and by the institutional review boards of all participating centers. Written informed consent was obtained from living patients; for deceased patients or when re-consent was impracticable, consent was waived in accordance with national regulations and the Declaration of Helsinki.

### Case eligibility

Eligible internal cases had histopathologic confirmation of PTPR according to the WHO classification criteria applicable at the time of diagnosis (2007, 2016, or 2021 editions), mandatory central neuropathology review of hematoxylin-and-eosin slides by D.C. (Berlin), comprehensive clinical, treatment, and survival data, and formalin-fixed paraffin-embedded tumor tissue with a histologically estimated tumor-cell content of at least 70% suitable for DNA extraction. Patients were excluded if they had a prior or concurrent CNS malignancy or if they had received antitumor treatment before tissue acquisition, except for diagnostic biopsy.

### Clinical annotation and follow-up

Clinical data were centrally collected in a standardized electronic case-report form and were quality-checked by L.L. and S.K. Baseline variables included age, sex, and Karnofsky performance score (KPS) before treatment. Treatment variables included the extent of resection, categorized as complete resection (no visible residual tumor on magnetic resonance imaging within 72 h after surgery), partial resection (any visible residual tumor), or biopsy performed for diagnostic intent only. Adjuvant radiotherapy and chemotherapy were also recorded. Follow-up was updated through December 31, 2023.

### Molecular cohort definition

Molecular quality control and subgroup verification were performed before inclusion in the analytic cohort. Adult samples were excluded if array quality was inadequate, normalization failed, or classifier-based assignment did not support a definitive PTPR molecular subgroup. All pediatric samples from the source cohort were retained.

For integrative unsupervised Uniform Manifold Approximation and Projection (UMAP) analyses, we used methylation data from 2,449 reference CNS tumor samples, generated on Illumina 450 K or EPIC arrays. These reference data were primarily derived from Capper et al. [11] through GEO accession GSE90496 and were processed as described in the original publication.

### DNA methylation processing and quality control

All DNA methylation processing and quality control procedures were performed in R version 4.5.1; package versions are listed in Supplementary Table S1. RnBeads version 2.26.0 was used for methylation preprocessing and quality control [17, 18]. Raw IDAT files from adult and pediatric tumors generated on EPIC v1.0 and 450 K arrays were imported with rnb.execute.import. Data from the different array types were imported separately and then combined with rnb.combine.arrays(type = "common") to retain CpG sites present on both platforms.

The standard quality control pipeline used rnb.run.qc with filtering.sex.chromosomes.removal = TRUE for autosomal analyses, filtering.cross.reactive = TRUE to remove probes known to cross-hybridize, and filtering.missing.value.quantile = 0.1 to remove probes with missing rates greater than 10% or probes failing internal RnBeads quality control metrics. Sample-level quality metrics, including beta-value densities and detection P values, were reviewed during quality control.

### Normalization, M-values, and batch correction

Preprocessing was performed with rnb.run.preprocessing. Normalization used method = "wm.dasen", which combines within-sample SWAN normalization with between-sample DASEN normalization. Background correction used bgcorr.method = "enmix.oob" for ENmix out-of-band correction. Methylation was reported as beta values, defined as β = M/(M + U + 100), where M is methylated intensity and U is unmethylated intensity. For downstream statistical modeling and differential methylation analysis, beta values were converted to M-values to improve statistical properties and homogeneity of variance.

Before combining adult and pediatric samples, batch effects and platform differences were assessed by principal-component analysis (PCA). Batch correction used ComBat [19] through the sva package [20], with array platform specified as the batch variable and age protected as a covariate.

### Methylation embedding and unsupervised analyses

For analyses integrating the study cohort with the reference CNS tumor data set, which included both 450 K and EPIC arrays, probes were first restricted to those common to both array platforms. ComBat was then used to adjust for array platform and processing batch before UMAP, so that the study and reference cohorts could be compared on the basis of biological methylation structure.

### UMAP and principal-component analyses

UMAP was performed with the R package umap version 0.2.10.0 [21]. To reduce noise and improve robustness, UMAP was computed from the top 15 principal components derived from the 10,000 most variable CpG probes. The UMAP settings were number of epochs = 10,000, number of neighbors = 7, minimum distance = 0.2, and random seed = 12,345. Sample clusters in UMAP plots were labeled with geom_label_repel from ggrepel version 0.9.6.

PCA was performed on centered and scaled M-values from the top 10% most variable CpG probes with PCAtools version 2.20.0. PCA plots were used to assess the influence of technical covariates. Unsupervised methylation heat maps were generated from the 2000 most variable CpG probes, with sample and probe order retained for supplemental tabulation.

### Copy-number analysis

Copy-number variation (CNV) analysis was performed with conumee2 version 2.1.2. Normal tissue control samples used to generate the baseline data were obtained from GEO accession GSE109381 and were preprocessed in the same way as the study cohort.

### Copy-number calling and segmentation

CNV annotation used CNV.create_anno, with array_type set to "EPIC" or "450 K" as appropriate for each cohort and genome = "hg19". Default binning parameters were used: bin_minprobes = 15, bin_minsize = 50,000, and bin_maxsize = 5e+06. CNV.fit was used to fit query intensities to the reference set, CNV.bin to combine probe intensities into bins, and CNV.segment to perform circular binary segmentation. Default segmentation parameters were alpha = 0.001, nperm = 50,000, min.width = 5, undo.splits = "sdundo", and undo.SD = 2.2. Genomic gains and losses were defined with an absolute log2-ratio threshold of 0.1. To test the robustness of broad arm-level CNV frequency estimates, sensitivity analyses used alternative absolute log2-ratio thresholds of 0.15 and 0.2.

Genomic CNV load was defined as the total number of bins with an absolute log2 ratio greater than 0.1. The association between age group and CNV load was evaluated with multivariable linear regression adjusted for molecular subgroup, array platform, sex, and tumor purity. Visual comparisons were shown as subgroup-stratified boxplots generated with ggpubr version 0.6.2. Age-group comparisons in very small subgroup strata were treated descriptively; formal within-subgroup significance testing was restricted to subgroup strata with sufficient pediatric representation.

### Focal and gene-level copy-number analysis

Segmentation files from conumee2 (.seg files) were analyzed with Genomic Identification of Significant Targets in Cancer (GISTIC) 2.0 version 2.0.23 through the GenePattern server [22], using the hg19 genome build. Significant arm-level amplifications and deletions were defined by a q-value less than 0.25. Default settings were used for focal length cutoff (0.98) and confidence level (0.75). Candidate driver genes within significant peaks were annotated from the GISTIC output.

For gene-level CNV analyses, bin-level log2 copy-number ratios were annotated to genes on hg19 using the mean bin ratio. Gene-level copy-number differences between age groups were assessed with Wilcoxon tests and Benjamini–Hochberg false discovery rate (FDR) correction. Results were visualized as heat maps stratified by molecular subgroup and age group with ComplexHeatmap version 2.24.1 [23].

### Differential methylation and functional annotation

Differentially methylated probes were identified with multivariable linear regression models implemented in limma version 3.64.3 [24]. The model included array platform, sex, PTPR subgroup, tumor purity, and age group, with age group as the main term of interest. P values were adjusted for multiple testing by the Benjamini–Hochberg FDR method. Significant differentially methylated probes were defined as probes with an FDR less than 0.05 and an absolute mean beta-value difference of at least 0.1, calculated as pediatric minus adult. To assess robustness against subgroup imbalance, a sensitivity analysis restricted the linear model to the PTPR-B subgroup. Heat maps of age-associated differentially methylated probes displayed the top 100 probes ranked by adjusted significance.

### Genomic context and pathway enrichment

Significant differentially methylated probes were mapped to hg19 genomic coordinates and annotated to CpG island contexts (island, shore, shelf, or open sea) and gene regions (TSS1500, TSS200, 5'UTR, first exon, body, 3'UTR, or intergenic) using the Illumina array manifest. Enrichment by genomic context was evaluated with Fisher's exact test to estimate odds ratios and 95% confidence intervals, followed by FDR correction. Pathway enrichment used the methylRRA algorithm to correct for probe-number bias. Analyses were performed separately for hypermethylated and hypomethylated promoter-associated probes, defined as probes in TSS200 or TSS1500 regions, against the Gene Ontology databases.

### Mitotic-clock and tumor microenvironment analyses

Mitotic age was estimated with the R package EpiMitClocks. epiTOC1, epiTOC2, and epiTOC3 were used as hypermethylation-based mitotic-clock models derived from the epiTOC framework [25], and HypoClock was included as a hypomethylation-based mitotic-like clock [26]. These scores were interpreted as proxies for the cumulative proliferative history of the tumor. epiTOC2 intrinsic rate score (epiTOC2 irS) was the prespecified primary mitotic-clock endpoint; the other clocks were considered supportive.

### Mitotic-clock models and correlations

Clock score comparisons were visualized with subgroup-stratified boxplots. Subgroup strata with very small pediatric cell counts were shown descriptively and were not emphasized for inference. Raw P values from within-subgroup Wilcoxon tests and Benjamini–Hochberg FDR values were calculated for all clock models. For each clock score, multivariable linear regression was used to estimate the independent association with age group, adjusting for molecular subgroup, array platform, sex, and tumor purity derived from MDBrainT2. Associations of epiTOC2 irS and epiTOC3 irS with genomic CNV load and estimated tumor purity were evaluated with Pearson correlation analyses stratified by subgroup, with Spearman analyses used as sensitivity checks.

### Methylation-based cell deconvolution

Cell type deconvolution was performed with two complementary approaches. First, deconvR version 1.14.2 was used with the comprehensive human methylome reference atlas from Moss et al. [27], using 450 K beta values subset to probes common to EPIC data. Sample beta values were used as input, and the default non-negative least-squares model was applied. Second, MDBrainT2 (R package MDBrainT version 0.1.0), which is designed for CNS tumor microenvironment deconvolution across 13 major CNS cell types, was applied with its non-negative least-squares algorithm.

Estimated cell fractions were obtained for all MDBrainT cell types. Age-group comparisons were performed within molecular subgroup strata with Wilcoxon tests and Benjamini–Hochberg FDR correction. Complete estimates were tabulated, and the main display was restricted to cell types selected after multiple testing correction. To account for potential confounding, associations between age group and cell fractions were also evaluated with multivariable linear regression models.

### Survival and statistical analysis

Progression-free survival was summarized descriptively with Kaplan–Meier curves. Time was measured from diagnosis to progression or last follow-up; patients without progression were censored at last follow-up. Because pediatric follow-up was sparse, pediatric-versus-adult survival comparisons were considered descriptive and were not used for inferential testing. All statistical analyses were performed in R version 4.5.1 with the packages listed in Supplementary Table S1. P values or FDR-adjusted P values less than 0.05 were considered statistically significant, except for GISTIC2.0 analyses, for which q values were used.

## Results

### Analytic cohort and methylation subgroups

The final analytic cohort included 86 histologically confirmed PTPR tumors, comprising 62 (72.1%) adult and 24 (27.9%) pediatric cases. Baseline clinical and technical characteristics are summarized in Table 1 and Supplementary Table S2. The sample selection diagram began with 94 patients, including 70 adults and 24 pediatric cases; eight adult samples, comprising six internal and two external cases, were excluded because of failed initial array quality control (n = 1), failed normalization by the greedy-cut algorithm (n = 3), or lack of definitive PTPR subgroup assignment (n = 4). The final adult cohort contained 19 internally collected and 43 external tumors, and the pediatric cohort contained 24 external tumors. All 24 pediatric samples passed quality control, leaving 86 tumors for core molecular analyses (Supplementary Fig. 1). Follow-up for progression-free survival was available for 55 patients (64%) overall, including 49 of 62 (79%) adults and 6 of 24 (25%) pediatric patients, and was therefore used only descriptively (Supplementary Fig. 1 and Supplementary Fig. 12).

**Table 1:** Baseline characteristics and data availability of the adult and pediatric PTPR cohorts

Characteristics	Adult Group (Age ≥18 years, N=62)	Pediatric Group (Age <18 years, N=24)
Clinical follow-up available, N (%)	49/62 (79%)	6/24 (25%)
Missing, N (%)	13/62 (21%)	18/24 (75%)
Follow-up in months, median (range)	63 (3–196)	29 (4–133)
Cohort composition		
Sex, N (%)		
Male	25 (40%)	13 (54%)
Female	37 (60%)	11 (46%)
PTPR Subgroup, N (%)		
PTPR-A	23 (37%)	2 (8%)
PTPR-B	39 (63%)	22 (92%)
Clinical variables		
Karnofsky Performance Score, N (%)		
≥ 90	11 (18%)	NA
< 90	8 (13%)	NA
NA	43 (69%)	24 (100%)
Extent of Resection, N (%)		
Biopsy	6 (10%)	NA
Partial resection	16 (26%)	NA
Complete resection	14 (22%)	NA
NA	26 (42%)	24 (100%)
Adjuvant Radiotherapy, N (%)		
Yes	16 (26%)	NA
No	20 (32%)	NA
NA	26 (42%)	24 (100%)
Data Availability, N (%)		
Methylation array (QC passed)	62 (100%)	24 (100%)
CNV derivation success	62 (100%)	24 (100%)
Cell deconvolution success	62 (100%)	24 (100%)

*NA* denotes not available, *PTPR* papillary tumors of the pineal region, *QC* quality control; and *CNV* copy-number variation. Several clinical variables were systematically unavailable in the pediatric cohort and are therefore presented descriptively rather than used for adult-versus-pediatric inference

### Methylation data quality

The retained methylation data showed acceptable technical quality after preprocessing. Normalized beta-value density profiles were stable across samples, and all samples retained for analysis exceeded the required probe detection threshold after one initial low-quality sample had been removed (Supplementary Fig. 2A, B). PCA showed that the strongest structure in the first two principal components was related to molecular subgroup rather than to technical covariates. Age group, molecular subgroup, platform, sex, and processing batch are shown in Supplementary Fig. 3A–E, respectively. Quantitative tests for the first two principal components are provided in Supplementary Table S10.

### Age group and methylation subgroup

**Fig. 1:**
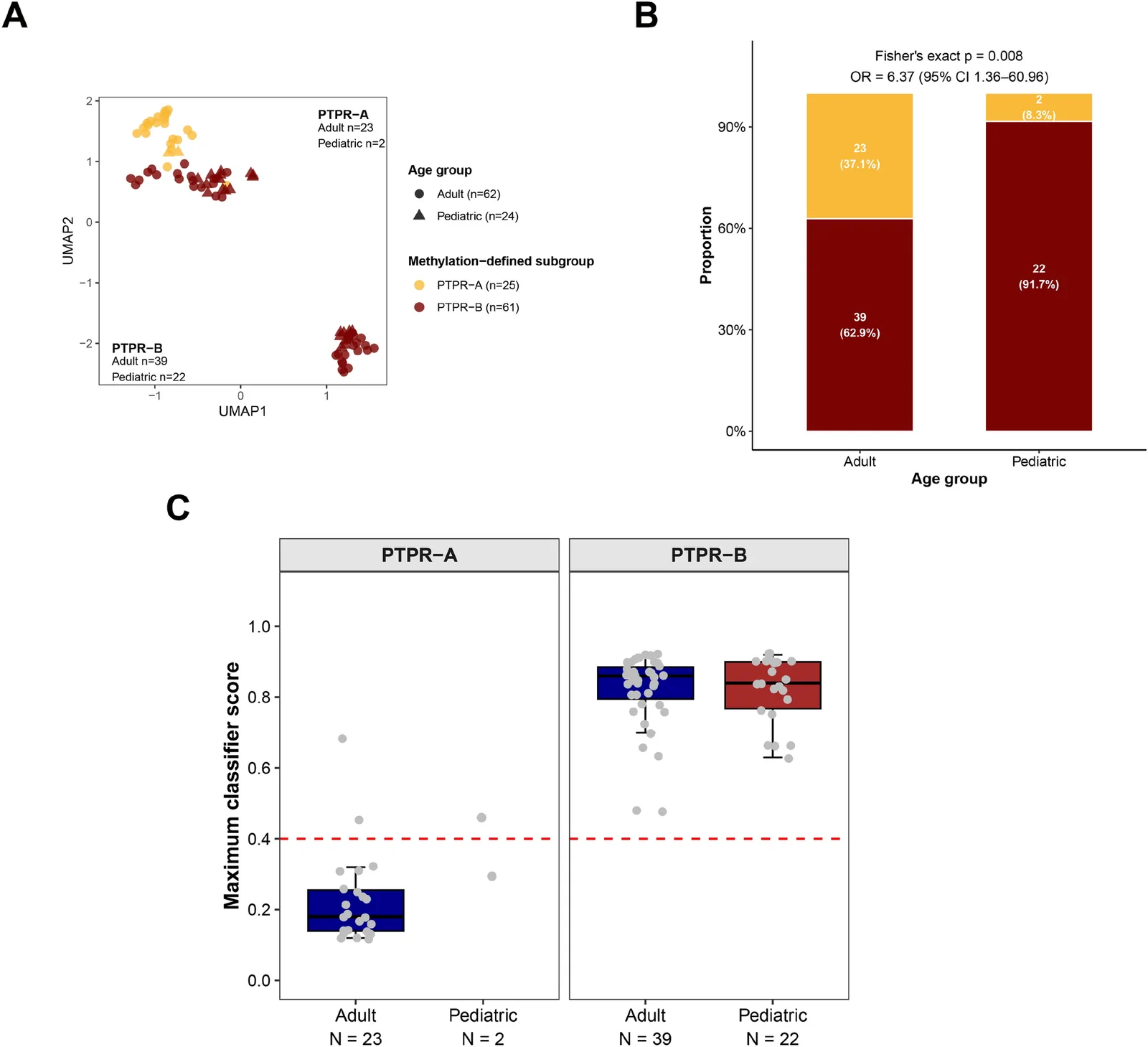
Molecular subgroup structure and cohort composition of adult and pediatric papillary tumors of the pineal region (PTPR). **A** Unsupervised Uniform Manifold Approximation and Projection (UMAP) of DNA methylation profiles from the final analytic cohort (n = 86), computed from the top 15 principal components derived from centered and scaled M-values for the top 10,000 most variable CpG probes. Points are colored by methylation-defined subgroup and shaped by age group. UMAP settings were number of neighbors, 7; minimum distance, 0.2; and random seed, 12,345. The embedding is descriptive; adjusted age effects are evaluated in Table 2 and Fig. 5. **B** Distribution of PTPR-A and PTPR-B by age group. Pediatric tumors were enriched for PTPR-B (22 of 24 tumors, 91.7%), whereas adult tumors included both PTPR-A (23 of 62, 37.1%) and PTPR-B (39 of 62, 62.9%) (Fisher's exact test, P=0.008; odds ratio, 6.37; 95% CI 1.36 to 60.96). **C** Maximum classifier scores from the Brain Tumor Classifier version 11b4, shown by age group and subgroup. The dashed line indicates the 0.4 diagnostic reliability threshold. Pediatric PTPR-A contains only two tumors and is shown descriptively. Lower scores in some PTPR-A tumors should be interpreted cautiously; platform composition and tumor purity are shown in Supplementary Figs. 3C and 3E, but this panel does not test those mechanisms directly

Unsupervised methylation analysis did not separate tumors into age-defined clusters. Instead, tumors were primarily organized by the established methylation-defined subgroups PTPR-A and PTPR-B (Fig. 1A). The pediatric cohort was strongly enriched for PTPR-B, with 22 of 24 tumors (91.7%) assigned to PTPR-B and only two assigned to PTPR-A. The adult cohort contained 23 PTPR-A tumors (37.1%) and 39 PTPR-B tumors (62.9%) (Fig. 1B). Classifier confidence was generally adequate for PTPR-B but was weaker in PTPR-A, particularly in the context of the very small pediatric PTPR-A subgroup (Fig. 1C). Therefore, age-associated comparisons across the full cohort were interpreted with subgroup imbalance in mind, and PTPR-B analyses were considered the most interpretable subgroup-restricted comparison.

Reference embedding supported diagnostic consistency of the final analytic set. In the global CNS methylation reference space, study samples mapped to the expected PTPR neighborhood rather than to unrelated CNS tumor classes (Supplementary Fig. 5A). A zoomed local view confirmed integration of adult and pediatric samples within the PTPR-A and PTPR-B molecular domains (Supplementary Fig. 5B). Descriptive progression-free survival curves are shown only for clinical context because outcome data were limited in pediatric cases: the analysis included 49 adults and six pediatric patients, with 21 adult events and 28 adult censored observations and three pediatric events and three pediatric censored observations (Supplementary Fig. 12).

### Age-associated DNA methylation

We next asked whether age-associated methylation differences were detectable after accounting for subgroup composition. Broad methylation summaries did not show a simple global age-related methylation shift. Mean global methylation was similar between age groups (P = 0.63), and average methylation levels across all CpGs, enhancer-associated CpGs, and promoter-associated CpGs were also similar (Supplementary Fig. 6A–D). The corresponding median differences and confidence intervals are provided in Supplementary Table S8.

### Adjusted differential methylation

**Fig. 2:**
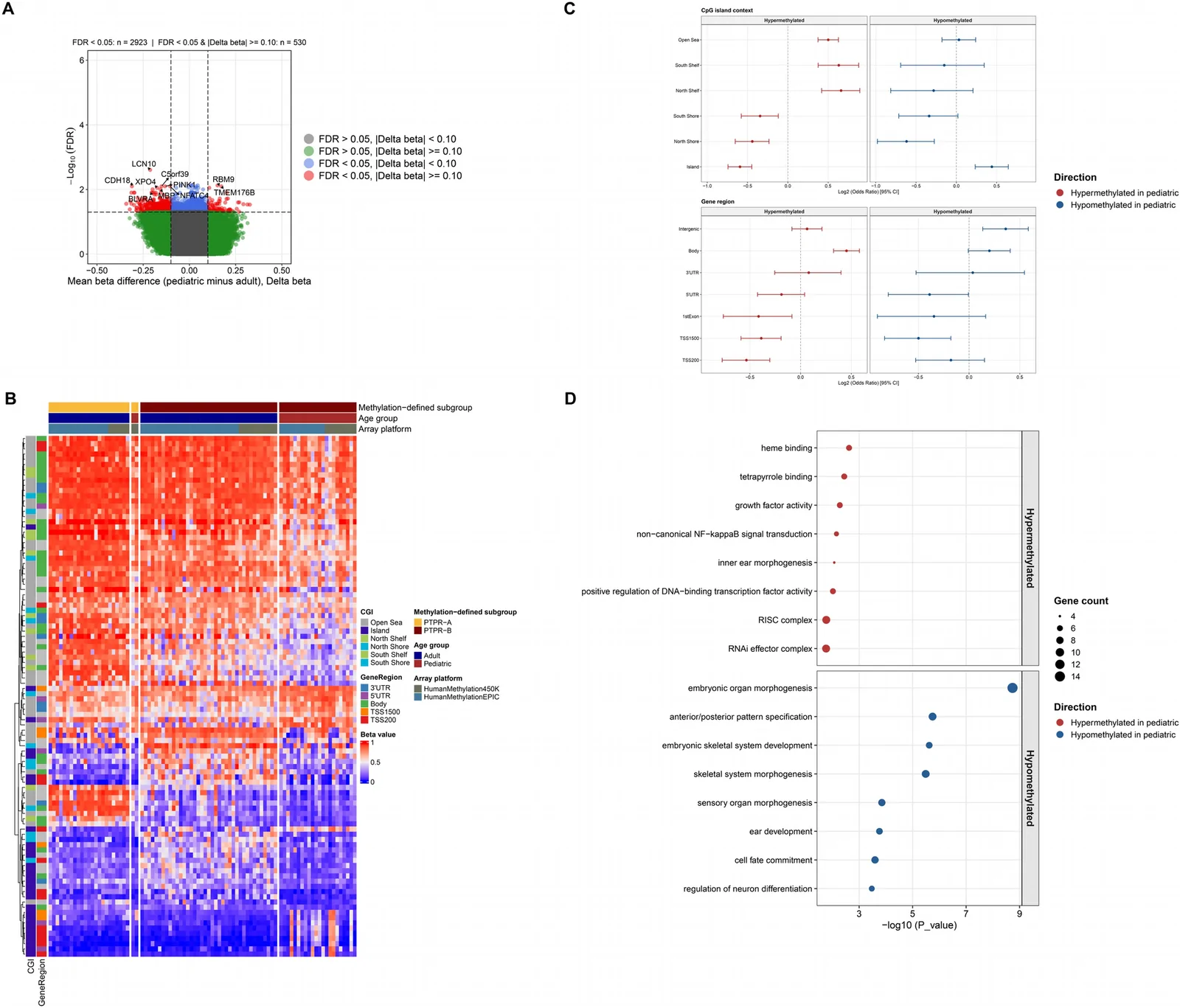
Age-associated differential DNA methylation and genomic context in PTPR. **A** Volcano plot of differentially methylated probes from a multivariable limma model of M-values with age group as the main term and adjustment for subgroup, platform, sex, and tumor purity. The x axis shows the mean beta-value difference (pediatric minus adult), and the y axis shows the -log10 false discovery rate (FDR) after Benjamini–Hochberg correction. A total of 2,923 probes met FDR<0.05, and 530 also met the combined threshold of FDR<0.05 and absolute beta difference of at least 0.1. **B** Heat map of the 100 leading FDR-significant differentially methylated probes, with samples ordered by subgroup and then by age group to display age-associated patterns while limiting subgroup-driven confounding. CpG island relationship and functional gene region annotations are shown. **C** Enrichment of age-associated probes by CpG island context and functional gene region. Odds ratios and 95% confidence intervals were calculated with Fisher's exact tests relative to the tested probe universe after quality control and are shown separately for hypermethylated and hypomethylated loci. **D** Pathway enrichment among promoter-linked differentially methylated probes mapping to TSS200 or TSS1500 regions. The direction of differential methylation is defined as pediatric minus adult; thus, 'hypermethylated' pathways denote loci with higher methylation in pediatric tumors, while 'hypomethylated' pathways denote loci with higher methylation in adult tumors. Enrichment was calculated with methylRRA to account for probe-number bias per gene

In contrast, the prespecified adjusted differential methylation analysis identified a restricted but measurable age-associated signal. A total of 2,923 probes met the prespecified FDR threshold, and 530 probes also met the combined statistical and effect-size threshold (Fig. 2A; complete results in Supplementary Table S4). The unsupervised methylation heat map showed that the strongest methylation structure was dominated by subgroup rather than by age (Supplementary Fig. 4). The corresponding probe and sample ordering are provided in Supplementary Table S3. The heat map of the leading age-associated differentially methylated probes showed within-subgroup age-associated methylation patterns, although age groups were not completely separated (Fig. 2B).

### Genomic context and pathway enrichment

Age-associated differentially methylated probes were not uniformly distributed across genomic contexts. Enrichment analyses showed differences by CpG island context and functional gene region (Fig. 2C), with numerators, denominators, and enrichment statistics provided in Supplementary Table S12. Promoter-linked pathway analysis identified developmental and regulatory terms among loci affected by age-associated promoter methylation, but these findings were interpreted as hypothesis-generating because methylation enrichment alone does not establish transcriptional activation or repression (Fig. 2D; Supplementary Table S5). In adjusted models across key molecular endpoints, the methylation signature represented by PC1 remained associated with age group in the full cohort and in the PTPR-B-only sensitivity analysis (Table 2, Fig. 5A, B, and Supplementary Fig. 13).

**Table 2:** Multivariable linear regression models evaluating the independent association between age group and key molecular endpoints

Outcome	Outcome scale	Pediatric-versus-adult coefficient	95% confidence interval	P-value	FDR-adjusted P-value	Complete-case number	Dropped number	Dropped samples
Genomic CNV load	Number of altered bins	− 579.77	[− 1269.31, 109.77]	0.098	0.098	86	0	NA
epiTOC2 irS	Estimated stem cell divisions (irS)	233.69	[147.59, 319.8]	6.89e−07	**1.38e−06**	84	2	ER46; U1
Methylation signature (PC1)	Principal component 1 score from high-variance methylation probes	− 28.58	[− 35.69, − 21.47]	8.19e−12	**3.28e−11**	86	0	NA
Neutrophils fraction (MDBrainT2)	Estimated cell fraction	0.04	[0.02, 0.06]	8.43e−05	**0.000112**	86	0	NA

Each row reports the pediatric-versus-adult coefficient from a separate complete-case multivariable linear model adjusted for methylation-defined subgroup, array platform, sex, and tumor purity. CNV denotes copy-number variation; FDR, false discovery rate

### Copy-number landscape and CNV burden

**Fig. 3:**
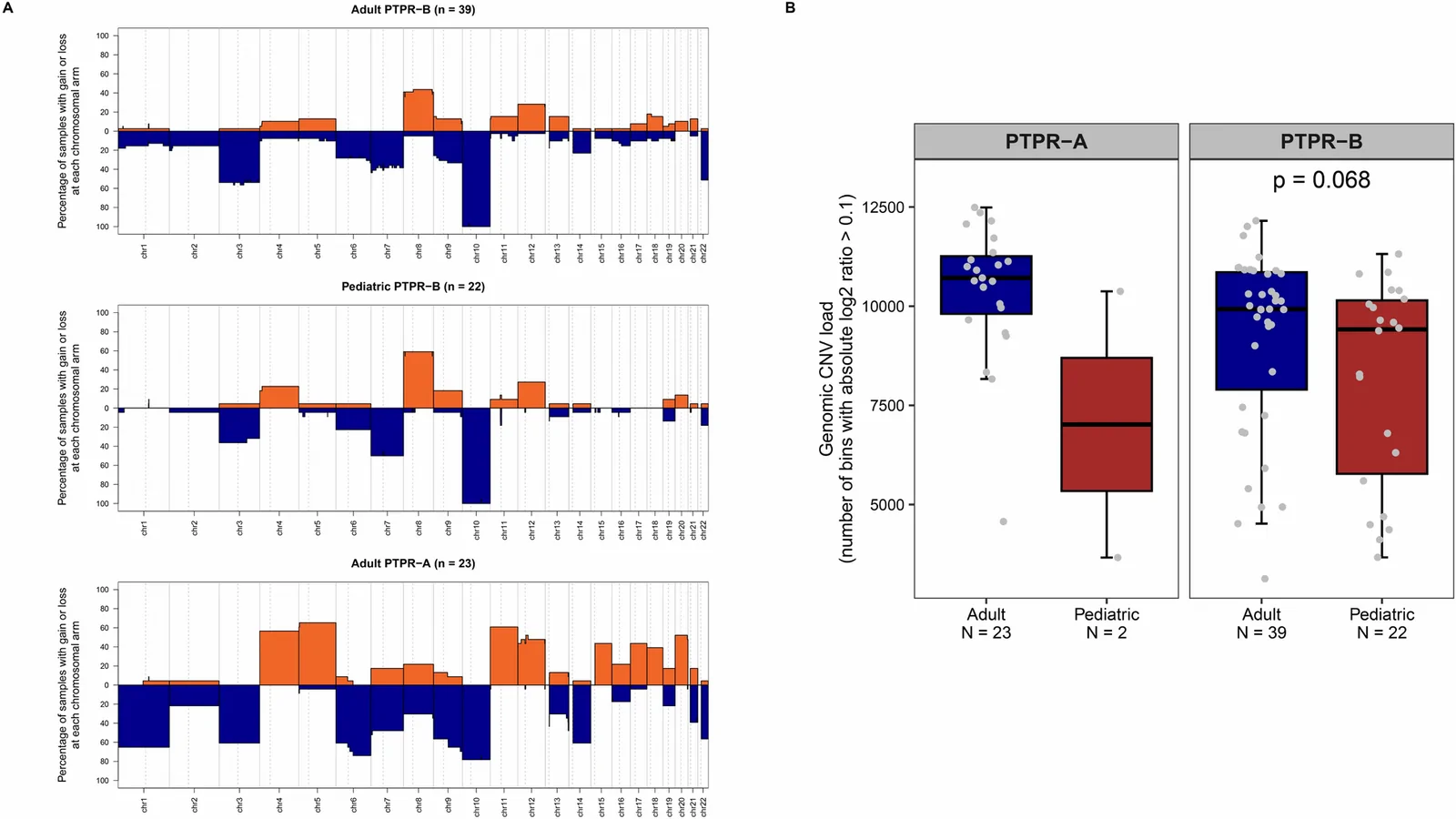
Copy-number landscape and genomic CNV load across age groups. **A** Chromosomal-arm copy-number frequency plots inferred from DNA methylation arrays with conumee. To limit subgroup confounding, frequencies are shown separately for adult PTPR-B (n = 39), pediatric PTPR-B (n = 22), and adult PTPR-A (n = 23). Gains are shown in orange and losses in blue using an absolute log2-ratio threshold of 0.1. Age-related CNV interpretation is restricted primarily to PTPR-B because pediatric PTPR-A included only two tumors. **B** Genomic CNV load, defined as the number of altered genomic bins with an absolute log2 ratio greater than 0.1, shown by age group and subgroup. The PTPR-A pediatric comparison is descriptive only. In the adjusted full cohort model, the pediatric-versus-adult coefficient for genomic CNV load was − 579.77 altered bins (95% CI − 1269.31 to 109.77; P=0.098; FDR=0.098), as reported in Table 2

Copy-number patterns were organized more strongly by methylation-defined subgroup than by age group. Adult PTPR-A showed a broader arm-level CNV landscape than PTPR-B, whereas adult and pediatric PTPR-B shared the major recurrent arm-level events, including prominent chromosome 10 loss (Fig. 3A). Chromosome 10 loss encompasses the PTEN locus at 10q23.31, although methylation-array-derived arm-level CNVs do not establish PTEN mutation, protein loss, or pathway activation. Frequencies and statistical estimates for chromosome arm events in adult and pediatric PTPR-B are provided in Supplementary Table S7. Given that only two pediatric tumors were assigned to PTPR-A, age-associated interpretation of CNV patterns was limited to the PTPR-B subgroup.

### Focal and gene-level copy-number findings

Focal and gene-level CNV analyses did not identify a robust age-defined CNV class. GISTIC2.0 did not yield a reproducible age-defined focal CNV pattern (Supplementary Fig. 7A–D). Pediatric peaks remained exploratory because of the limited sample size (Supplementary Table S13). Gene-level CNV heat maps showed subgroup-linked structure without a clear age-segregated amplification or deletion pattern (Supplementary Fig. 8 and Supplementary Table S6).

**Fig. 4:**
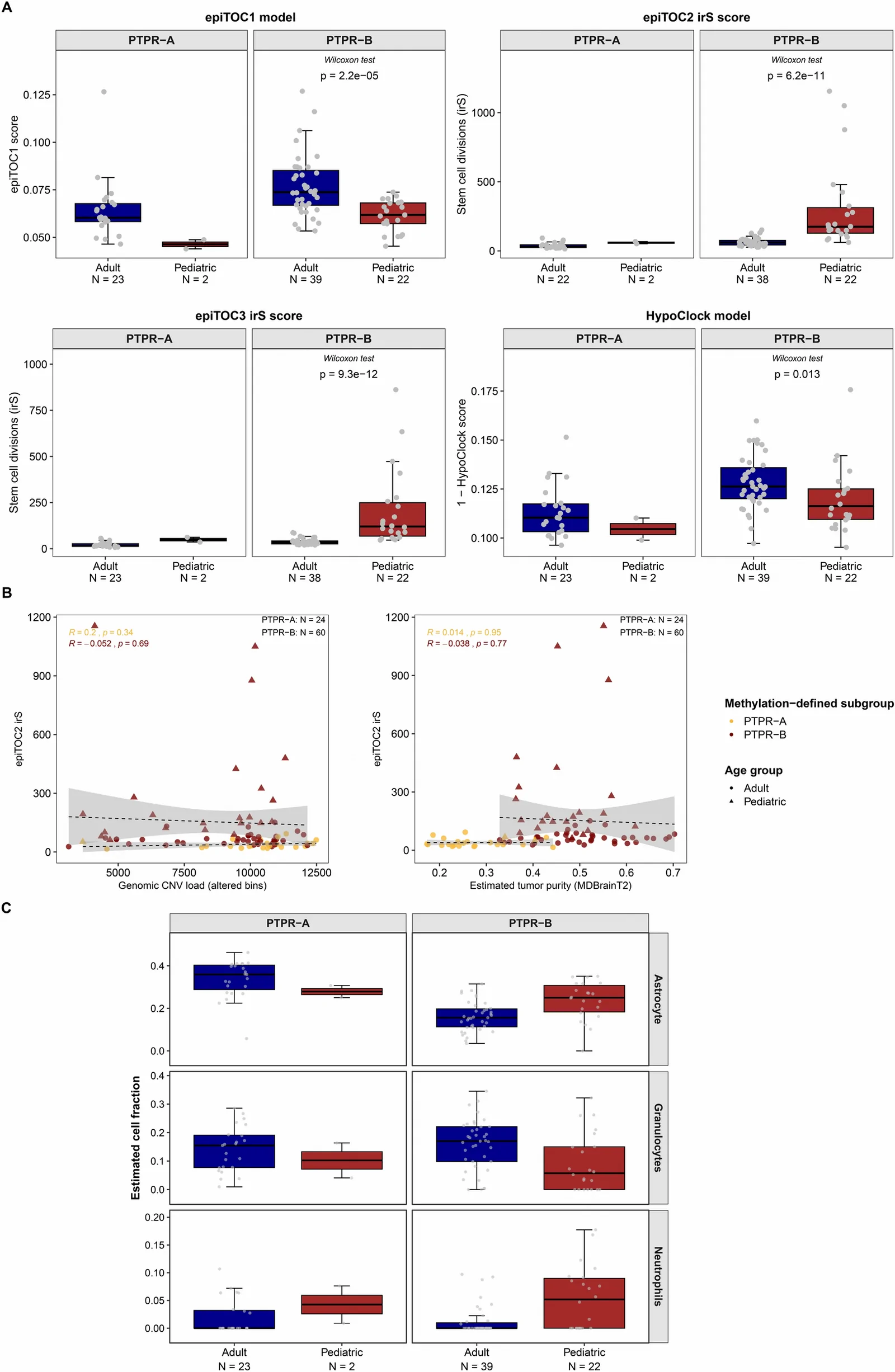
Mitotic-clock signatures and methylation-based microenvironment estimates. **A** Subgroup-stratified distributions of four epigenetic mitotic-clock scores (epiTOC1, epiTOC2, epiTOC3, and HypoClock), which are proxies for proliferative history. epiTOC2 intrinsic rate score (irS) was the prespecified primary mitotic-clock endpoint; the other clocks were supportive analyses. Pediatric PTPR-A (n = 2) is shown descriptively. Raw P values and FDR values are provided in Supplementary Table S9. **B** Correlations of epiTOC2 irS with genomic CNV load and MDBrainT2-derived tumor purity. Pearson correlation coefficients and P values are shown by subgroup; Spearman sensitivity analyses are provided in Supplementary Table S11. These correlations are exploratory and were not used to infer causality. **C** Selected MDBrainT2-derived cell-fraction estimates, shown by subgroup and age group. Complete cell type estimates and multiple testing results are provided in Supplementary Table S14. Cell-fraction estimates are exploratory and require orthogonal validation

Broad CNV findings were stable in sensitivity analyses across alternative CNV-calling thresholds (Supplementary Fig. 10A–C). These results supported the robustness of recurrent arm-level patterns but did not indicate a distinct age-specific CNV architecture (Fig. 4).

### Adjusted CNV burden

**Fig. 5:**
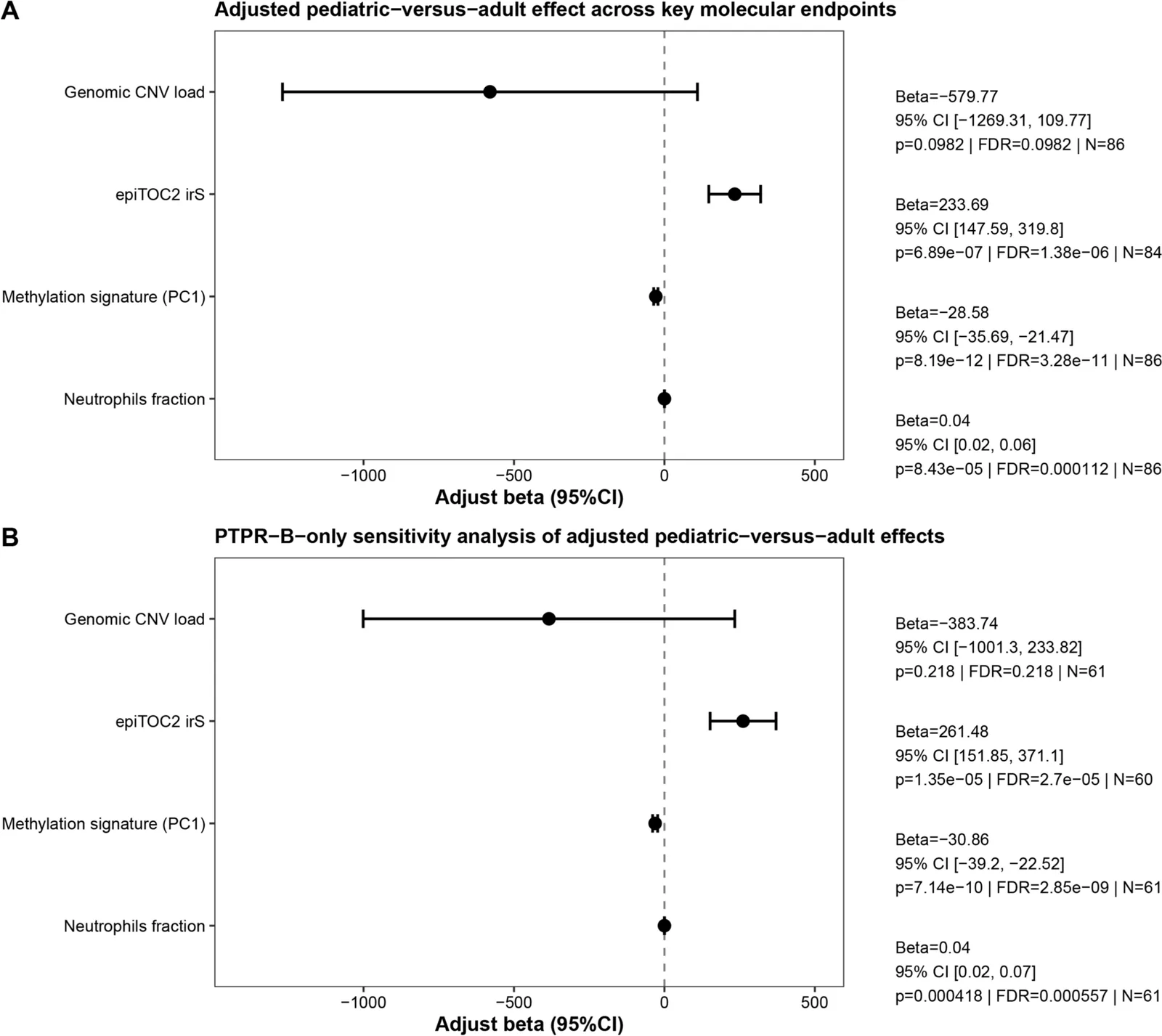
Adjusted pediatric-versus-adult effects across molecular endpoints. **A** Forest plot of separate complete-case multivariable models in the full analytic cohort, adjusted for subgroup, platform, sex, and tumor purity. Endpoints are genomic CNV load, epiTOC2 irS, methylation signature represented by PC1, and neutrophil fraction. Coefficients, 95% CI, raw P values, FDR-adjusted P values, and complete-case sample sizes are displayed. **B** PTPR-B-only sensitivity analysis using the same model structure. PTPR-A was not modeled separately because only two pediatric PTPR-A tumors were available

Quantitative CNV burden analyses were consistent with the aforementioned conservative interpretation. Subgroup-stratified boxplots showed a descriptive PTPR-A comparison and a weak age-associated difference within PTPR-B (Fig. 3B). In the prespecified adjusted model, however, age group was not an independent predictor of genomic CNV load in the full cohort (Table 2 and Fig. 5A). The PTPR-B-only model yielded a similar conclusion (Fig. 5B), and standardized coefficients across molecular endpoints likewise did not support a dominant age effect on CNV load (Supplementary Fig. 13).

### Mitotic-clock signatures and tumor microenvironment

We then examined whether age-associated differences extended to proliferation-linked methylation measures. Across four epigenetic mitotic-clock models, the most coherent age-associated pattern was observed for epiTOC2 intrinsic rate score, the prespecified primary mitotic-clock endpoint, whereas epiTOC1, epiTOC3, and HypoClock were supportive and not fully concordant (Fig. 4A; Supplementary Table S9). Pediatric tumors tended to have higher epiTOC2 intrinsic rate scores than adult tumors within PTPR-B, whereas the PTPR-A comparison remained descriptive because only two pediatric PTPR-A tumors were available. The adjusted full cohort model supported an age-associated epiTOC2 signal (Table 2, Fig. 5A, and Supplementary Fig. 13), with a directionally similar assessment in PTPR-B-only sensitivity analysis (Fig. 5B).

### Mitotic-clock correlates

The mitotic-clock signal was not explained simply by genomic instability or tumor admixture. Correlations between epiTOC2 intrinsic rate score and genomic CNV load or MDBrainT2-derived tumor purity were modest and inconsistent across subgroups (Fig. 4B). Supportive epiTOC3 analyses showed similarly exploratory relationships (Supplementary Fig. 11). Correlation sensitivity analyses are provided in Supplementary Table S11. These analyses assessed plausibility and were not used to infer causality.

### Methylation-based microenvironment estimates

Methylation-based cell deconvolution suggested a limited age-associated tumor-microenvironmental signal across selected MDBrainT2-derived cell fractions (Fig. 4C). Complete cell type estimates and multiple testing-adjusted results are provided in Supplementary Table S14. Analyses based on the two prespecified reference frameworks are shown in Supplementary Fig. 9A, B. Concordance and direction changes are summarized in Supplementary Table S15 and Supplementary Table S16. In the prespecified adjusted model, the neutrophil fraction was the most defensible microenvironmental association (Table 2, Fig. 5A, and Supplementary Fig. 13), but the broader deconvolution pattern remained exploratory and did not support a strong age-dependent immune-remodeling program.

## Discussion

In this study, we examined whether PTPR show an age-related molecular architecture or whether adult and pediatric tumors remain organized within the established PTPR methylation framework. The central observation was that age did not create a separate methylation class. This conclusion is anchored in prior work showing PTPR as a molecular entity [10], as a CNS methylation class [11], and as a tumor type with PTPR-A and PTPR-B molecular subgroups [7, 12]. The present data extend that framework by showing that adult and pediatric tumors map within the same diagnostic space, arguing against pediatric PTPR as a separate diagnosis solely on the basis of age. Yet molecular equivalence should not be inferred: pediatric cases were enriched in PTPR-B, so age, subgroup, and cohort composition remain closely linked and require subgroup-aware interpretation.

The most reproducible age-associated signal was epigenetic rather than broadly genomic. Global methylation summaries were similar between age groups, but adjusted probe-level analyses identified a restricted set of differentially methylated CpG sites enriched in defined genomic contexts and promoter-associated pathways. Age-associated epigenetic and molecular patterns in glioma [13, 14], and age-linked molecular structure in medulloblastoma [15] make such a signal biologically plausible in PTPR. However, it should be interpreted as a tissue-level epigenetic association, not as proof of altered transcriptional output. Methylation arrays do not measure RNA expression, chromatin accessibility, or protein abundance, and enrichment from promoter-associated CpGs can overstate functional directionality. Therefore, the defensible conclusion is narrower: within the shared PTPR framework, age is associated with measurable epigenetic differences not fully explained by PTPR molecular subgroup.

By contrast, copy-number analyses provided little support for a robust age-defined genomic class. The dominant CNV structure remained subgroup-linked, with adult PTPR-A showing broader arm-level alterations and PTPR-B showing recurrent events shared by adult and pediatric tumors. The absence of an independent age effect on genomic CNV load is relevant because copy-number burden is often used as a surrogate for genomic instability. In this cohort, genomic CNV load did not provide an age-specific measure of genomic instability. The observed age associations were concentrated in methylation-derived measures and do not establish a distinct age-specific CNV architecture, consistent with an integrated WHO classification that is not based on age alone [3].

The mitotic-clock analyses add a second layer. The epiTOC2 intrinsic rate score showed the clearest age-associated pattern, particularly within the interpretable PTPR-B comparison. Because epiTOC was developed as an epigenetic mitotic-clock proxy [25] and later mitotic-like clocks capture different aspects of proliferative history [26], these results should not be equated with mitotic count, Ki-67 labeling, or clinical aggressiveness. The adjusted association and weak relationships with CNV load or estimated purity support the robustness of the epiTOC2 signal within this data set. The epiTOC2 result remains a methylation-derived proxy of proliferative history and does not establish differences in clinical aggressiveness. Differences in growth kinetics, treatment sensitivity, or recurrence risk require validation in independent cohorts with orthogonal assays.

The tumor microenvironment results were limited. Methylation-based deconvolution suggested at most a modest age-associated signal, with neutrophil fraction showing the most defensible adjusted association. This observation is hypothesis-generating. The methylation atlas used for deconvolution provides broad human cell type reference profiles [27], but bulk methylation deconvolution remains affected by tumor purity, reference set composition, and incomplete representation of relevant CNS and inflammatory cell states. In rare tumors such as PTPR, these constraints are amplified by small subgroup sizes. Thus, microenvironment findings require orthogonal validation with RNA sequencing, immunohistochemistry, spatial profiling, or single-cell approaches before clinical inference.

Several limitations should temper interpretation. The study was retrospective, with nonuniform treatment, tissue handling, array platforms, and follow-up across cohorts. The pediatric PTPR-A cohort was too small for reliable age-by-subgroup inference, leaving PTPR-B as the only credible subgroup-restricted comparison. In addition, the available data did not support robust analysis of the recently proposed PTPR-B1 and PTPR-B2 subdivisions. Follow-up was available for 49 of 62 adults and 6 of 24 pediatric patients. Extent of resection and adjuvant radiotherapy were unavailable for every pediatric case, and dissemination status was not systematically captured across cohorts. A pooled prognostic or treatment-response model would therefore be dominated by adult observations and vulnerable to selection bias and informative missingness. Progression-free survival remained descriptive. Tumor purity, cell fractions, and mitotic-clock scores were inferred computationally, and these tools have not been calibrated specifically in PTPR. Public data were essential for studying this rare tumor but limited control over preanalytic variables. These limitations define the evidentiary boundary of the study.

Chromosome 10 loss warrants specific translational consideration because it encompasses PTEN at 10q23.31. Prior PTPR studies have identified PTEN deletions or mutations together with PI3K/AKT/mTOR pathway activation [28]. Individual reports have described durable radiographic responses to everolimus in recurrent PTPR with chromosome 10 and PTEN loss or a PTEN R130Q alteration [29, 30]. These cases provide a biological rationale for molecular profiling in recurrent disease and prospective evaluation of pathway-directed therapy. They do not establish efficacy or a predictive biomarker. The present cohort lacks matched PTEN sequencing, PTEN immunohistochemistry, phosphoprotein assays, and treatment-response data. Chromosome 10 loss alone should therefore not guide treatment selection.

**Fig. 6:**
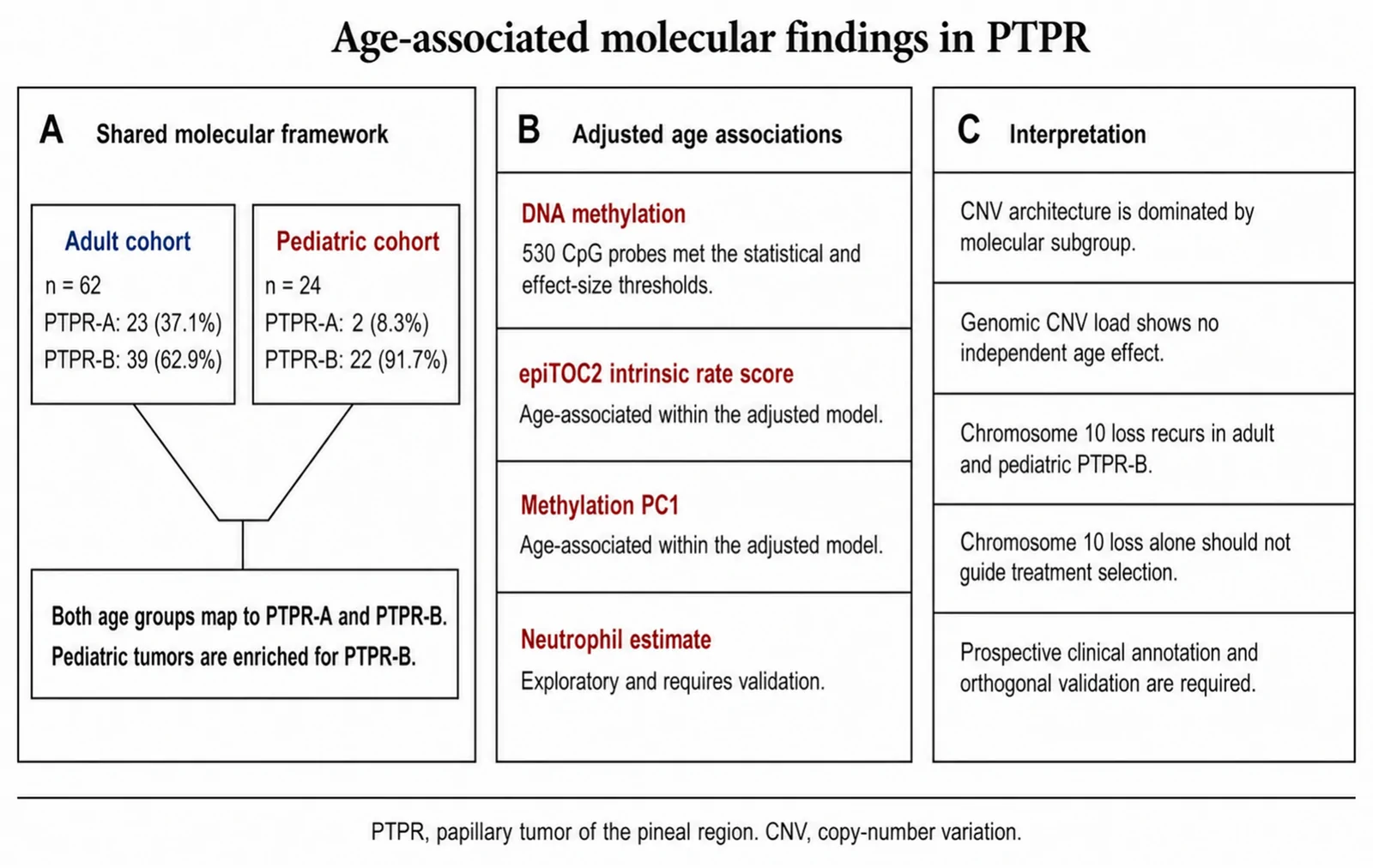
Schematic summary of the principal age-associated molecular findings in PTPR. Adult and pediatric tumors shared the PTPR-A/PTPR-B methylation framework, with enrichment of PTPR-B in the pediatric cohort. Adjusted analyses identified age associations for CpG methylation, epiTOC2 intrinsic rate score, and PC1. Copy-number architecture remained dominated by molecular subgroup, and genomic CNV load showed no independent age effect. Chromosome 10 loss recurred in adult and pediatric PTPR-B. The molecular findings are hypothesis-generating and require prospective clinical annotation and orthogonal validation

The clinical implication is conservative. DNA methylation profiling is well supported for CNS tumor classification [11] and has become central to contemporary PTPR molecular characterization [7]. The present data do not support age-based treatment stratification or prognostic counseling. Current treatment evidence for PTPR remains largely retrospective and literature review based [6, 9]. Age can be treated as a biologically informative covariate within an integrated molecular framework. Recent pediatric pineal-tumor literature similarly supports molecularly stratified study designs rather than age-only categories [16]. Future studies should prioritize international prospective registries with standardized recording of extent of resection, dissemination at diagnosis and recurrence, radiotherapy, systemic therapy, progression criteria, and survival outcomes. Multi-omics and tissue-based validation should accompany these clinical data. In summary, adult and pediatric PTPR share the established PTPR-A/PTPR-B framework, and pediatric tumors, particularly within PTPR-B, show age-associated epigenomic heterogeneity. These findings define testable biological hypotheses for future clinically annotated cohorts. A schematic summary of the main findings is provided in Fig. 6.

## Supplementary Information


Supplementary Material 1.
Supplementary Material 2.


## Data Availability

Publicly available datasets analyzed in this study were obtained from GEO (accession numbers GSE254031 and GSE90496). The data generated and analyzed by the authors during the current study are included in this article and its supplementary materials. Additional data are not publicly available due to patient privacy and ethical restrictions but are available from the corresponding author upon reasonable request.
