# Supplementary material for: Age-associated epigenomic heterogeneity in papillary tumors of the pineal region: a multicenter YoungNOA investigation
Source: Acta Neuropathol Commun. 2026 Aug 28;14:180. doi: 10.1186/s40478-026-02411-x (PMC13525666; doi:10.1186/s40478-026-02411-x)
Supplement: Supplementary file 1 [file 40478_2026_2411_MOESM2_ESM.docx]

**Supplementary materials**

**Supplementary Figure 1. Cohort flow diagram and sample selection.** The retrospective source cohort included 94 patients (70 adults and 24 pediatric patients). Eight adult samples were excluded because of failed initial array quality control (n = 1), failed normalization by the greedy-cut algorithm (n = 3), or lack of definitive PTPR subgroup assignment (n = 4). All pediatric samples passed quality control. The final analytic cohort comprised 86 tumors (62 adult and 24 pediatric tumors). Progression-free survival data were available for 55 patients, including 49 adults and six pediatric patients; survival analyses were therefore descriptive.

**Supplementary Figure 2. DNA methylation quality control metrics.** (A) Normalized beta-value density profiles after preprocessing. Similar density shapes support acceptable normalization but do not exclude residual platform or batch effects. Complementary technical assessments are shown in Supplementary Figures 3C and 3E. (B) Proportion of CpG probes meeting the detection threshold of P<0.01 in each sample. The horizontal line indicates the minimum pass rate required for downstream analysis. One sample failed initial array quality control; all retained samples exceeded the threshold.

**Supplementary Figure 3. Principal-component analysis of technical and biological covariates.** PCA was performed on scaled M-values for the top 10% most variable CpG probes in the final analytic cohort (n = 86). Identical axis scaling is used across panels. Points are colored by (A) age group, (B) molecular subgroup, (C) array platform, (D) sex, and (E) processing batch. The first two principal components were dominated primarily by subgroup, with weaker age-related structure. Quantitative associations with PC1 and PC2 are reported in Supplementary Table S10. Platform was retained as the primary technical adjustment variable in downstream multivariable models.

**Supplementary Figure 4. Methylation heat map of high-variance CpG probes.** Heat map of the 2,000 most variable CpG probes, with rows representing CpG probes and columns representing samples. Samples were ordered by unsupervised hierarchical clustering. Top annotations show methylation-defined subgroup, age group, and array platform; side annotations show CpG island context and functional gene region categories. Clustering used Euclidean distance and complete linkage. Probe and sample ordering are provided in Supplementary Table S3.

**Supplementary Figure 5. Reference methylation embedding of study samples.** (A) Global UMAP projection of study samples (n = 86) within the reference space of 91 established CNS tumor classes. Study samples mapped to the expected PTPR region; unrelated reference classes are shown for context. (B) Local PTPR view confirming integration of adult and pediatric tumors within the PTPR-A and PTPR-B methylation domains. Counts are shown next to local PTPR clusters.

**Supplementary Figure 6. Global methylation summaries.** (A) Mean global methylation per sample; distributions were similar between age groups (Wilcoxon test, P=0.63). (B through D) Average methylation across all CpGs, enhancer-associated CpGs, and promoter-associated CpGs. Median differences and 95% confidence intervals are provided in Supplementary Table S8. These analyses summarize broad methylation levels and do not replace probe-level differential methylation analysis.

**Supplementary Figure 7. GISTIC2.0 analysis of focal copy-number alterations.** Focal amplifications (red) and deletions (blue) are shown separately for adults (A and C; n = 62) and pediatric patients (B and D; n = 24). The x axis shows q values, and the green vertical line marks the genome-wide significance threshold (q = 0.25), applied identically across panels. Significant peaks are annotated with cytobands and candidate genes. Pediatric focal peaks are exploratory because of limited sample size. Peak genes are listed in Supplementary Table S13.

**Supplementary Figure 8. Gene-level copy-number alteration heat map.** Gene-level log2 copy-number ratios are shown for the top 50 selected genes ranked by FDR in the differential gene-level CNV analysis. Rows represent genes and columns represent samples ordered as in the subgroup-based methylation heat map. Top annotations indicate age group and methylation-defined subgroup; side annotations indicate chromosomal location and direction of effect. Rows and columns were not reclustered so that the prespecified ordering was preserved. Complete results are provided in Supplementary Table S6.

**Supplementary Figure 9. Sensitivity analysis of methylation-based cell deconvolution.** Estimated cell type abundances are shown with two reference frameworks: (A) the standard Human Cell Type Methylation Atlas and (B) the blood-specific FlowSorted.Blood.450k reference. Boxplots are stratified by molecular subgroup. Agreement between frameworks is summarized in Supplementary Table S15, and direction changes across reference frameworks are listed in Supplementary Table S16.

**Supplementary Figure 10. Sensitivity analysis of broad CNV frequency calls.** Recurrent chromosomal-arm gains and losses were re-evaluated with three absolute log2-ratio thresholds: 0.1, 0.15, and 0.2. Percentages of samples with gains or losses are shown for (A) adult PTPR-B, (B) pediatric PTPR-B, and (C) adult PTPR-A. Pediatric PTPR-A was not modeled separately because only two tumors were available. These panels test robustness and are not independent biological results.

**Supplementary Figure 11. Supportive correlation analysis for epiTOC3 irS.** Scatter plots show correlations of epiTOC3 irS with genomic CNV load and MDBrainT2-derived tumor purity. Pearson correlation coefficients and P values are shown by subgroup; Spearman sensitivity analyses are provided in Supplementary Table S11. This analysis complements the epiTOC2 irS correlations shown in Figure 4B and is exploratory.

**Supplementary Figure 12. Progression-free survival by age group.** Kaplan-Meier curves compare adults (n = 49) and pediatric patients (n = 6) with available follow-up. The number-at-risk table is shown below the x axis, and tick marks denote censored observations. Because only six pediatric patients had follow-up, this analysis is descriptive and does not support definitive inferential comparison. Adult follow-up included 21 events and 28 censored observations; pediatric follow-up included three events and three censored observations.

**Supplementary Figure 13. Standardized pediatric-versus-adult effects across molecular endpoints.** Standardized coefficients and 95% confidence intervals are shown from separate complete-case multivariable linear models adjusted for subgroup, platform, sex, and tumor purity. Endpoints include genomic CNV load, epiTOC2 irS, methylation signature (PC1), and neutrophil fraction. Corresponding unstandardized estimates and model details are provided in Table 2.

**Supplementary Table S1. R package versions and key reference resources.**

**Supplementary Table S2. Expanded per-sample clinical metadata, technical covariates, and data availability indicators.**

**Supplementary Table S3. High-variance CpG probes and sample ordering used for hierarchical clustering.**

**Supplementary Table S4. Complete multivariable differential DNA methylation results for the pediatric-versus-adult comparison.**

**Supplementary Table S5. Functional pathway enrichment analysis of age-associated promoter-linked differentially methylated probes.**

**Supplementary Table S6. Complete gene-level copy-number alteration results.**

**Supplementary Table S7. Chromosome arm-level copy-number alteration frequencies and 95% confidence intervals in adult and pediatric PTPR-B tumors.**

**Supplementary Table S8. Median differences and 95% confidence intervals for global methylation summary comparisons between pediatric and adult tumors.**

**Supplementary Table S9. Raw P values and FDR-adjusted P values for subgroup-stratified comparisons of mitotic-clock models between pediatric and adult tumors.**

**Supplementary Table S10. Quantitative associations of biological and technical covariates with the first two principal components.**

**Supplementary Table S11. Pearson and Spearman sensitivity analyses for subgroup-stratified correlations of epiTOC2 irS and epiTOC3 irS with genomic CNV load and estimated tumor purity.**

**Supplementary Table S12. Numerators, denominators, and enrichment statistics for age-associated differentially methylated probes by CpG island context and gene region.**

**Supplementary Table S13. Focal amplification and deletion peak genes identified by GISTIC2.0 analysis.**

**Supplementary Table S14. Complete subgroup-stratified MDBrainT2 cell deconvolution results for pediatric-versus-adult comparisons across cell types.**

**Supplementary Table S15. Correlations between the standard Human Cell Type Methylation Atlas and FlowSorted.Blood.450k reference frameworks for each cell type.**

**Supplementary Table S16. Direction changes of age-associated cell type effects across deconvolution reference frameworks.**
